# Temperature–humidity index monitoring during two summer seasons in dairy cow sheds in Mugello (Tuscany)

**DOI:** 10.1007/s00484-023-02510-7

**Published:** 2023-08-01

**Authors:** Alessandro Messeri, Marco Mancini, Riccardo Bozzi, Silvia Parrini, Francesco Sirtori, Marco Morabito, Alfonso Crisci, Gianni Messeri, Alberto Ortolani, Bernardo Gozzini, Simone Orlandini, Luca Fibbi, Simone Cristofori, Daniele Grifoni

**Affiliations:** 1 LaMMA Consortium–Laboratory of Monitoring and Environmental Modelling for Sustainable Development, 50019 Sesto Fiorentino, Florence, Italy; 2 Fondazione per il Clima e la Sostenibilità, Via G. Caproni, 50146 Florence, Italy; 3 Associazione Meteo Professionisti (AMPRO), via Francesco Morandini 30, 00142 Roma, Italy; 4 Department of Agriculture, Food, Environment and Forestry (DAGRI), University of Florence, 50144 Florence, Italy; 5 Institute of Bioeconomy, National Research Council (IBE-CNR), 50019 Florence, Italy

**Keywords:** Animal welfare, Heat waves, Climate change, Heat stress, Animal behaviors

## Abstract

Many studies have reported that the impact of high temperatures affects physiology, welfare, health, and productivity of farm animals, and among these, the dairy cattle farming is one of the livestock sectors that suffers the greatest effects. The temperature–humidity index (THI) represents the state of the art in the evaluation of heat stress conditions in dairy cattle but often its measurement is not carried out in sheds. For this reason, the aim of this study was the monitoring of the THI in three dairy cattle farms in Mugello (Tuscany) to understand its influence on dairy cows. THI values were calculated using meteorological data from direct observation in sheds and outdoor environments. Data relating to the animal’s behavior were collected using radio collars. The Pearson test and Mann–Kendall test were used for statistical analysis. The results highlighted a significant (*P* < 0.001) upward trend in THImax during the last 30 years both in Low Mugello (+ 1.1 every 10 years) and in High Mugello (+ 0.9 every 10 years). In Low Mugello sheds, during the period 2020–2022, more than 70% of daytime hours during the summer period were characterized by heat risk conditions (THI > 72) for livestock. On average the animals showed a significant (*P* < 0.001) decrease in time spent to feeding and rumination, both during the day and the night, with a significant (*P* < 0.001) increase in inactivity. This study fits into the growing demand for knowledge of the micro-climatic conditions within farms in order to support resilience actions for protecting both animal welfare and farm productivity from the effects of climate change. This could also be carried out thanks to estimation models which, based on the meteorological conditions forecast, could implement the thermal stress indicator (THI) directly from the high-resolution meteorological model, allowing to get a prediction of the farm’s potential productivity loss based on the expected THI.

## Introduction

It is known that climate change is causing an increase in the average temperature of the planet (NASA 2020, Masson-Delmotte et al. 2021) and 2021 was the seventh consecutive year in the period, between 2015 and 2021 (WMO 2021), in which the global temperature was 1 °C above the threshold of pre-industrial mean values (1850–1900). Europe is one of the areas of the planet most sensitive to climate change which is manifested above all by an increase in summer temperatures associated with an increase in the frequency and intensity of heat waves (Morabito et al. 2017; Vitali et al. 2020; Maggiolino et al. 2022). The Copernicus Climate Change Service reports in August 2022, a global surface air temperature 0.3 °C higher than the 1991–2020 average, and in particular in Europe during the period June–August 2022, the temperature was 0.4 °C higher over 2021, the hottest summer on record. Heat stress in animals is one of the major climate change impacts on livestock raised in both intensive and extensive production systems (Polsky and Von Keyserlingk. 2017; Pasqui and Edmondo 2019; Thornton et al. 2022).

The global dairy cattle industry is one of the most affected livestock sectors from heat stress (Gauly and Ammer 2020). In the last years, there was an increasing demand for milk (Polsky and von Keyserlingk 2017), due to the constant increase in the world population especially in emerging economies and with a consequent increase in the number of cattle raised, which has led to increased interest in the influence of heat stress on this sector. Numerous studies show that particularly high temperatures and therefore heat stress conditions during the lactation phase determine an increase in the metabolic heat produced by animals with a decrease in milk production and a worsening of its quality (Tao et al. 2020), with a decrease in the content of fats and proteins in conjunction with an increase in somatic cells (Chebel et al. 2004; Pinto et al. 2020). In addition to the problem related to milk production, the Italian Minister of Agriculture, Food Sovereignty and Forests (MASAF) also highlights problems regarding reproduction as well as animal welfare (MASAF 2023), an aspect that is increasingly requested by the consumer, as also reported by a recent study carried out in Ireland on final consumers (Hyland et al. 2022). Heat stress affects the basic behaviors which consequently can influence the welfare and production of the animal. Lacetera (2018) and Islam et al. (2021) revealed that heat stress can cause metabolic dysfunctions, oxidative stress, and immune suppression in large animals, generating infections and consequently altering the animal’s welfare and performance. In the breeding of dairy cows, to reduce the problem, with acclimatization (Nardone et al. 2010), the animals adopt strategies such as the reduction of feed intake leading to a reduction in rumination and the alteration of some physiological functions (reproduction and productive efficiency), to increase the maintenance metabolism (Acquilani et al. 2020) at the expense, however, of the energy balance which is negative (Soriani et al. 2013). This also leads to a general increase in the animal’s inactivity (Nordlund et al. 2019). A systematic review to assess the effect of heat stress on the behavior of lactating cows housed in compost barns (Frigeri et al. 2023) showed that heat stress generally promoted decreases in feed events and the time that cows spent lying down. On the contrary, the authors report an increase in events of visiting the water trough, the number of steps, agonistic behavior, and dyspnea.

These effects, until a few decades ago, were only manifested at low latitudes, where the persistence of high temperatures was frequent, now with climate change are also found at medium and high latitudes and in geographical areas where these climatic conditions were rare (Schüller et al. 2014).

The temperature and humidity index (THI), used to evaluate the conditions of well-being/heat stress for humans (Thom 1959), even considering only temperature and humidity, is currently the most used indicator in livestock (Pinto et al. 2020), particularly in dairy cattle (De Rensis et al. 2015; Ouellet et al. 2019). However, THI monitoring is not carried out on all dairy farms. Often, in small and medium-sized Italian farms (about < 100 and 100 ÷ 300 cows, respectively), equipped with sheds without cooling systems for animals, no THI registration is provided.

In Tuscany, the project, “The precision livestock farming systems in the management of dairy cattle breeding in Mugello to cope with climate change” (MILKLIMAT), aims to assess the impact of microclimatic conditions, in particular heat, on the health and performance of dairy cattle, to propose adaptation strategies that make it possible to protect both animal welfare and farms productivity.

The aim of this study was to monitor, during summer seasons, the THI values in three small/medium-sized dairy cow farms located in a Tuscan pre-Apennine valley (Mugello), to evaluate, if also in this area the climate change may generate or increase thermal discomfort in dairy cattle. This will allow providing a useful tool for breeders to plan heat risk mitigation interventions within the farm and thus safeguard animal welfare and milk production.

## Methods

### Study area

The study was carried out in three dairy farms, located in a Tuscany Apennine area (Mugello). The territorial layout of Mugello is characterized by an evident bipartition due to the Apennine: the southern part, Low Mugello (LM), a wide valley (average altitude close to 250 m a.s.l.) and the northern part, High Mugello (HM), a narrower valley (average altitude 350 m a.s.l.). LM and HM are exposed towards west–southwest and east–northeast, respectively; HM appears more protected by the Apennines against mild and humid western currents, but it is more exposed to cold eastern currents, the opposite occurs for LM.

**Fig. 1:**
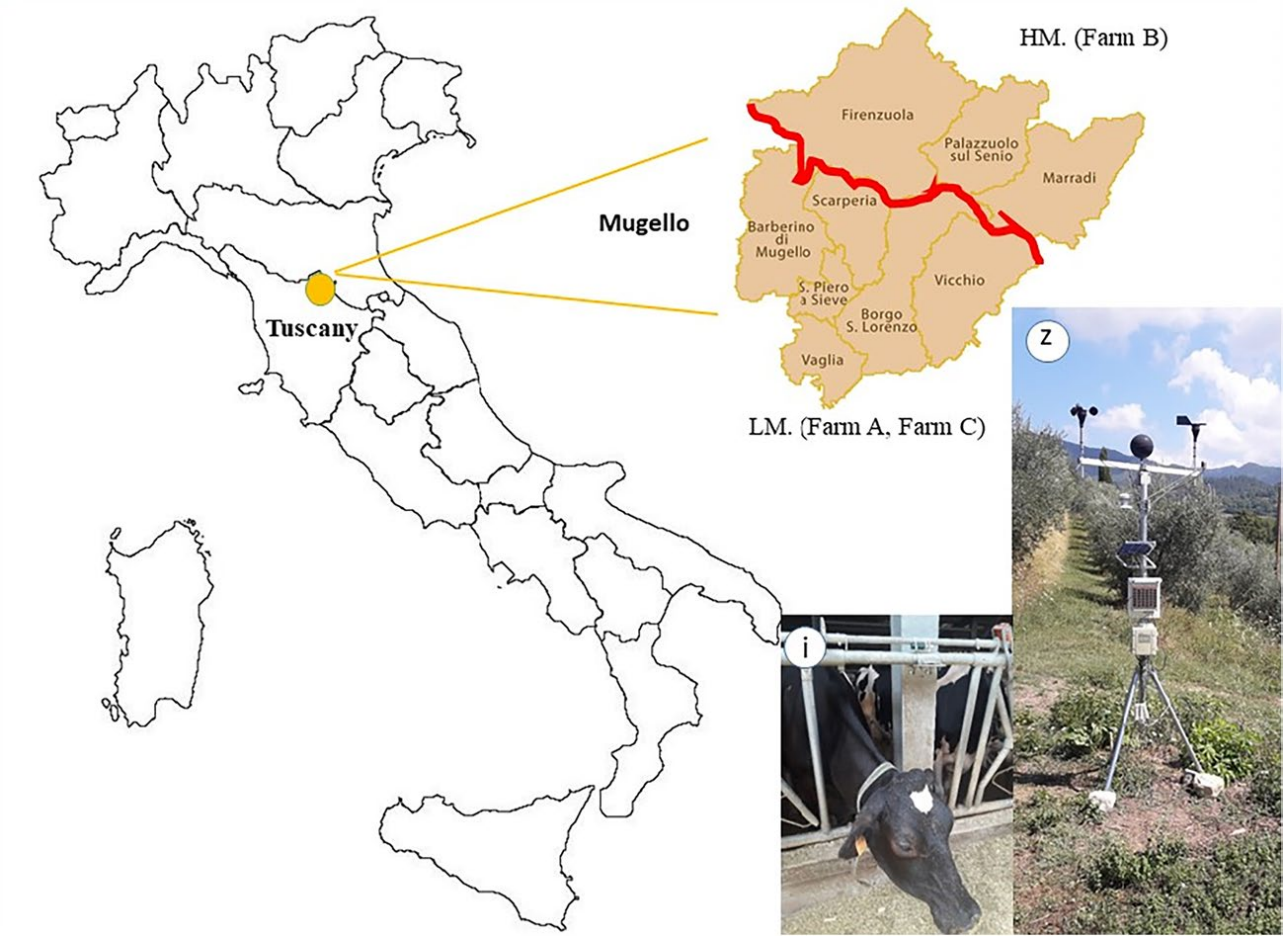
Study area: Mugello valley in Tuscany (Italy). LM, Low Mugello; HM, High Mugello; i, thermo-hygrometer installed in the dairy cow sheds; z, weather station located in one of the farms in LM

The other geographic variables, in addition to the exposition, i.e., latitude, longitude, and distance from the sea are similar between LM and HM. Two farms are in LM close to the locations of Vicchio (203 m a.s.l) and Luco di Mugello (306 m a.s.l.) (Farm A: Lat. 43° 57′ 49.392″ N, Long. 11° 26′ 48.048″ E and Farm C: Lat. 44° 0′ 30.528″ N, Long.11° 23′ 10.752″ E), one farm in HM around the location of Firenzuola (420 m a.s.l.) (Farm B: 44° 7′ 53.372″ N, 11° 21′ 38.2″ E) (Fig. 1). The altitude of the farms range between 260 (Farm A) and 575 m a.s.l. (Farm B).

### Farm management

The three farms have a total of about 400 lactating cows with an average daily milk production of 32 kg/head/day. The farming system showed only an isolated cover without walls with similar height (about 5.5 m). The cows were kept in a free stall consisting of a feed alley and staging area. The cows were absent from the pen during milking, for approximately 30 minutes, twice a day from 5:00 to 7:00 and 17:00 to 19:00.

### Data collection

#### Meteorological data from direct observation

Air temperature (°C) and relative humidity (%) were collected in the summer (June–August) for the period 2020–2022 inside three dairy cow sheds using thermo-hygrometer data loggers model Xtech RHT10 (Fig. 1(i)) with an hourly sampling interval. In particular, 3 thermo-hygrometers data logger were installed inside each shed at three different levels from the ground (0.5 m, 1.5 m, and 3 m) and an average value between the three heights was calculated. The three thermo-hygrometers were centrally located in the shed between the alley and the resting cubicles. Moreover, to monitor the outdoor environmental conditions (however not frequented by animals), a complete weather station, model HOBO U30 NRC (Fig. 1(z)), and a thermo-hygrometer data logger shielded from radiation, were installed in LM and HM, respectively.

#### Meteorological data derived from spatialization

Since the time series thus collected were limited to only three summer seasons and therefore not being useful for outdoor environment climatological considerations, a research for meteorological weather stations with a long time series around the farms was carried out. The lack of such meteorological weather stations in the study area made it necessary to use outdoor spatialized data. LaMMA Consortium is the available database of meteorological parameters spatialized over Tuscany region.

The daily outdoor spatialized data were calculated using the all meteorological daily observations available for the Tuscany Region archived in the LaMMA database for the period 1991–2022. The weather stations used belong to the networks of Ufficio idrografico di Pisa, Genova, Parma, Bologna e Roma, Servizio Idrologico della Regione Toscana, ARSIA, Aeronautica Militare, LaMMA Consortium, UCEA, and other minor networks for a total of 965 termometric stations and 588 hygrometric stations. The spatialized meteorological parameters used in this study are maximum daily air temperature and average daily relative humidity.

The spatialization was performed using an improved version of the Daymet algorithm (Thornton et al. 1997). This algorithm generates a spatial interpolation of the meteorological variables using a DTM of the area of interest and the observations from a series of weather stations. The original algorithm has been calibrated for the Tuscany region, using a DTM at 250 m around Tuscany (in the area between 44.5719–42.1323 N latitude and 9.68646–12.474 W longitude) for the period 1995–2017 (17 years). The results of cross-validation for the calibration period show a mean error of − 0.04736 °C and − 0.08613% and mean absolute error of 1.35214 °C and 6.72616% for the maximum temperature and average relative humidity. For the location of Firenzuola (HM) and Borgo San Lorenzo (LM), the latter in an intermediate position between Vicchio and Luco di Mugello extracted the daily outdoor spatialized data of maximum air temperature and relative humidity of the summer, for the period 1991–2022.

#### Behavioral data of animals

The physiological and behavioral data of the animals were collected through the use of sensors within collars applied to dairy cow. The systems Cowscout used, together with the herd management system DairyPlan C21 (GEA Farm Technologies, Germany; manufactured by Nedap Livestock Management, the Netherlands), recorded 24/7 the data continuously to monitor cow activity by identifying varies movements. The sensors detect specific activities patterns, recording the time individual cows spend eating, ruminating and standing still (inactivity). Each behavior was expressed as frequency during the day. All these data were used to evaluate welfare/heat stress conditions of dairy cows based on THI changes inside sheds in the study period.

### Thermal stress indicator

The temperature–humidity index (THI) was used in this study to evaluate well-being/heat stress conditions of dairy cows according to the following formula (NRC 1971; Yan et al. 2021):

$$\textrm{THI}=\left(1.8\times \textrm{TA}+32\right)-\left(0.55-0.0055\times \textrm{RH}\right)\times \left(1.8\times \textrm{TA}-26\right)$$

where TA is the air temperature (°C) and RH is the relative humidity (%).

There are numerous THI thresholds used internationally to identify risk levels for dairy cows and in this study those provided by the Italian Ministry of Agriculture, Food Sovereignty and Forestry (MASAF) have been used; two classifications of risk can be used, the first with lower THI thresholds aimed to protect milk productivity, and the second with higher THI thresholds aimed to protect from cow mortality. Furthermore, each of the two classifications provides different thresholds for the daytime (07:00–20:00) and nighttime (20:00–07:00). In this work, daylight and nighttime thresholds (Table 1) to protect milk productivity were used.

**Table 1:** THI daylight and nighttime milk productivity thresholds for dairy cow. Modified by https://www.politicheagricole.it/flex/cm/pages/ServeBLOB.php/L/IT/IDPagina/6095

Risk class	Level	Daylight thresholds	Nighttime thresholds
1	No risk	THI ≤ 72	Ore THI ≤ 62
2	Low risk	72 < THI ≤ 78	Ore 62 < THI ≤ 68
3	Moderate risk	78 < THI < 84	Ore 68 < THI < 74
4	Alert	THI ≥ 84	Ore THI ≥ 74

The hourly daytime and nighttime THI was calculated inside the sheds (shed THI) using the data collected by the thermo-hygrometric data loggers for the three summer periods 2020–2022; for the same period, the corresponding outdoor (outdoor observed THI) data were calculated using values from the complete meteorological station and from the thermo-hygrometric data logger, respectively, in LM and HM. All hourly THI data were also classified according to the daylight and nighttime thresholds to protect milk productivity. The frequency distribution of hourly THI data together with the percentage of hours falling into each risk class for outdoor and inside sheds, daylight and nighttime, were calculated for each summer month. The outdoor observed THI daily maximum (outdoor observed THImax) was also calculated. Shed THI and outdoor observed THI on an hourly basis were also compared by means of a linear regression analysis.

The outdoor THImax was also calculated for the period 1991–2022 using the outdoor daily spatialized data (outdoor spatialized THImax) of Borgo San Lorenzo and Firenzuola.

Over the three summer seasons 2020–2022 a comparison between the outdoor spatialized THImax and the outdoor observed THI max was carried out to verify the degree of representativeness of the spatialized data for the two locations.

Outdoor spatialized THImax was aggregated over each summer and its trend in the period 1991–2022 was calculated for LM and HM; in addition, also, the number of summer days with outdoor spatialized THImax higher than the lower heat risk threshold (> 72) and then higher risk class (> 84) was calculated.

### Data analysis

Regarding climatic data, the significance of the trends was calculated using the Pearson test (Pearson 1990) where the distribution was parametric, while in the case of non-parametric distribution, the Mann–Kendall test was used (Mann 1945). The significant levels were set to *P* < 0.05 (*), *P* < 0.01 (**), and *P* < 0.001 (***). Graphs and tables were created with Microsoft Excel. As regards animal’s data, records of rumination, inactivity, and eating behavior were normally distributed. Data were analyzed under the following linear mixed model, using the R function *lmer* of *lm4* R package (Bates et al. 2015):

$${Y}_{ijkl}=\mu +{\textrm{THI}}_i+{F}_j+\left({\textrm{THI}}_i\ast {F}_j\right)+\left({A}_k\right|\ {F}_j\Big)+{e}_{ijkl}$$

where *Y* is the behaviors expressed as frequency/100 for ruminating, inactivity, and eating, *μ* is the overall mean, THI is the effect of the *i*th THI estimates, analyzed both as the average of diurnal THI (calculated from 7:00 AM to 7:00 PM), and the average of nocturnal THI (calculated from 8:00 PM to 6:00 AM) and expressed as continuous variables, *F* fixed effect of the jth farm (3 levels), *A* is the random effect of the *k*th animal, nested within the farm, and *e* is the random residual.

## Results

### Outdoor spatialized THI trend analysis over the period 1991–2022

**Fig. 2:**
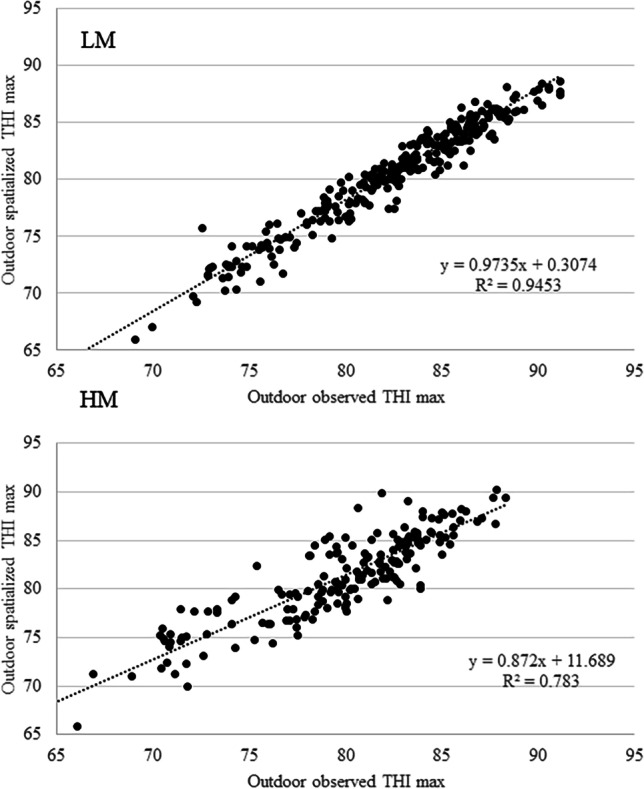
Correlation (*P* < 0.001) between outdoor spatialized THImax and outdoor observed THImax for LM and HM in the summer periods 2020–2022

In order to proceed to a climatological evaluation of the outdoor heat stress conditions of the study areas and considering the absence of meteorological stations with a long timeseries of data, it was necessary the use historical timeseries derived from spatialization procedure. In the period of contemporary availability of outdoor observed THImax and outdoor spatialized THImax (three summers), a comparison between them was carried out by means of a linear regression; a very good correlation was observed (Fig. 2) being statistically significant both for LM (*R*^2^ = 0.94, *P* < 0.001) and HM (*R*^2^ = 0.78, *P* < 0.001).

**Fig. 3:**
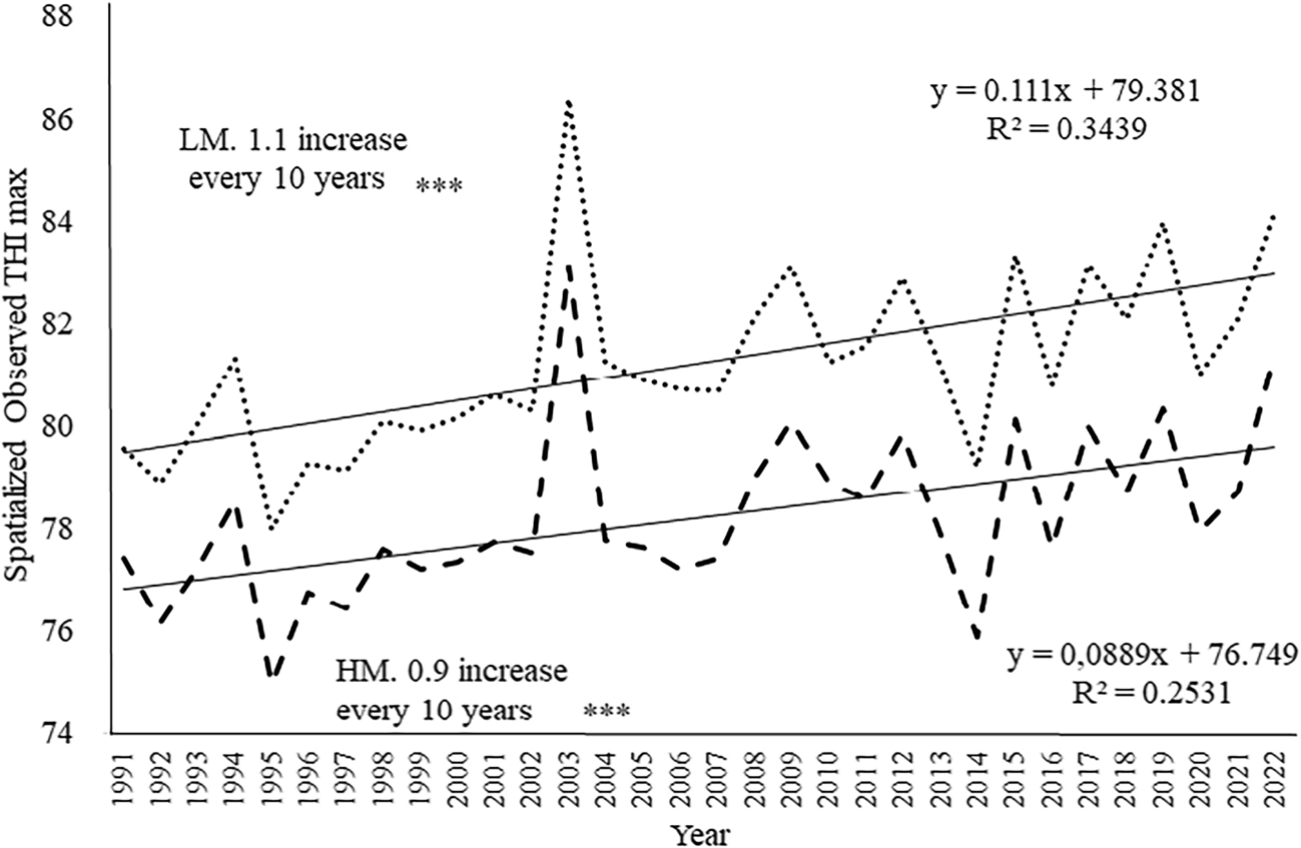
Trend of summer outdoor spatialized THImax during in the period 1991–2022. LM Borgo S. Lorenzo, point line; HM Firenzuola, dashed line. *P < 0.05, **P <0.01, and ***P < 0.001; n.s. not statistically significant

By using the outdoor spatialized THI, it was possible to calculate the possible trends of THI over the period 1991–2022. The summer outdoor spatialized THImax showed a highly significant (*P* < 0.001) upward trend in the last 30 years both in LM (+ 1.1 every 10 years) and in HM (+ 0.9 every 10 years) (Fig. 3).

**Fig. 4:**
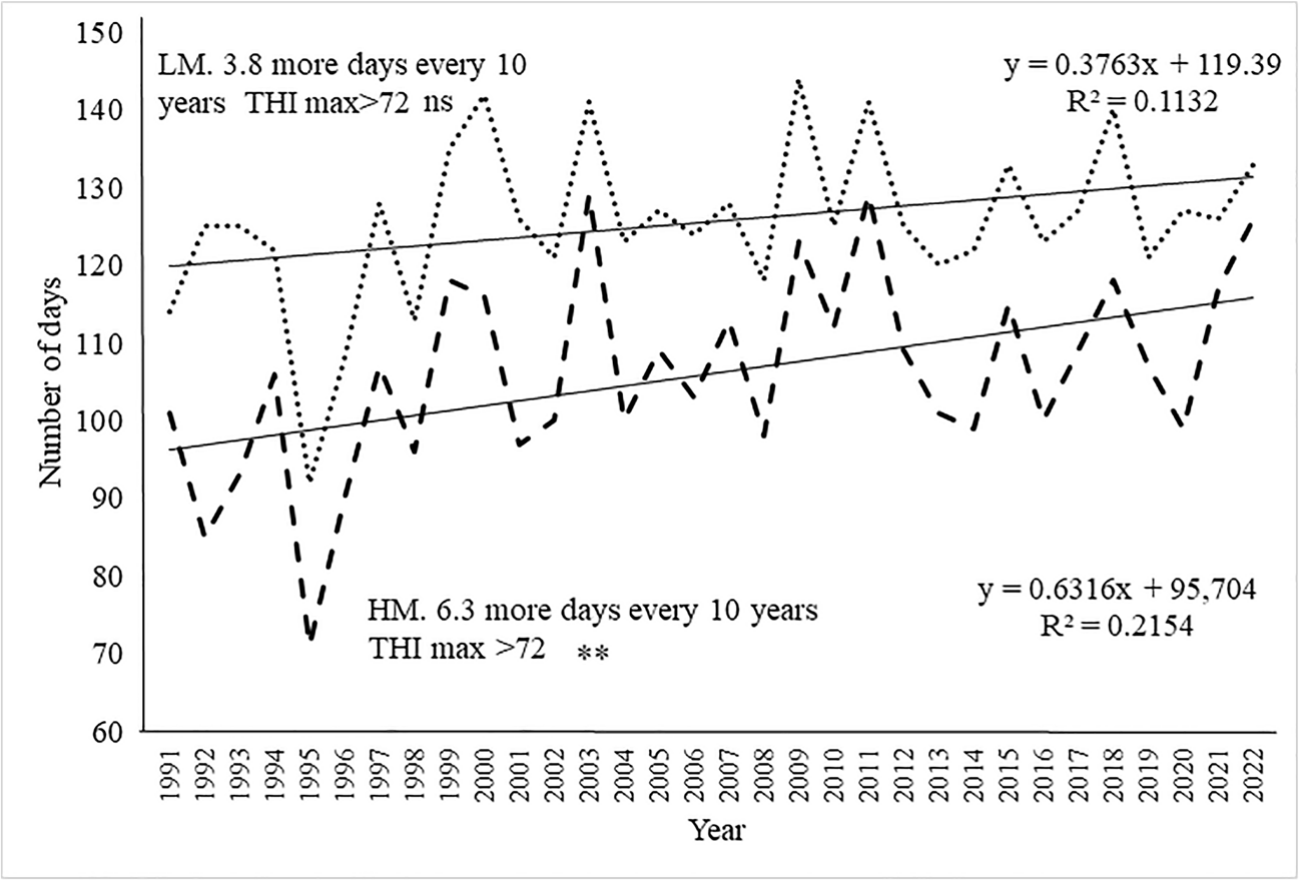
Number of summer days with outdoor spatialized THImax > 72 (risk classes 2, 3, and 4) in the period 1991–2022. LM Borgo S. Lorenzo, point line; HM Firenzuola, dashed line. *P <0.05, **P < 0.01, and ***P <0.001; n.s, not statistically significant

On the other hand, considering the number of days with THImax higher than the lower daytime risk threshold (> 72, classes 2, 3 and 4), the upward trend was statistically significant (*P* < 0.01) only in the HM with an increase of more than 6 days every 10 years (Fig. 4), against an upwards trend of 3.6 more days for LM.

**Fig. 5:**
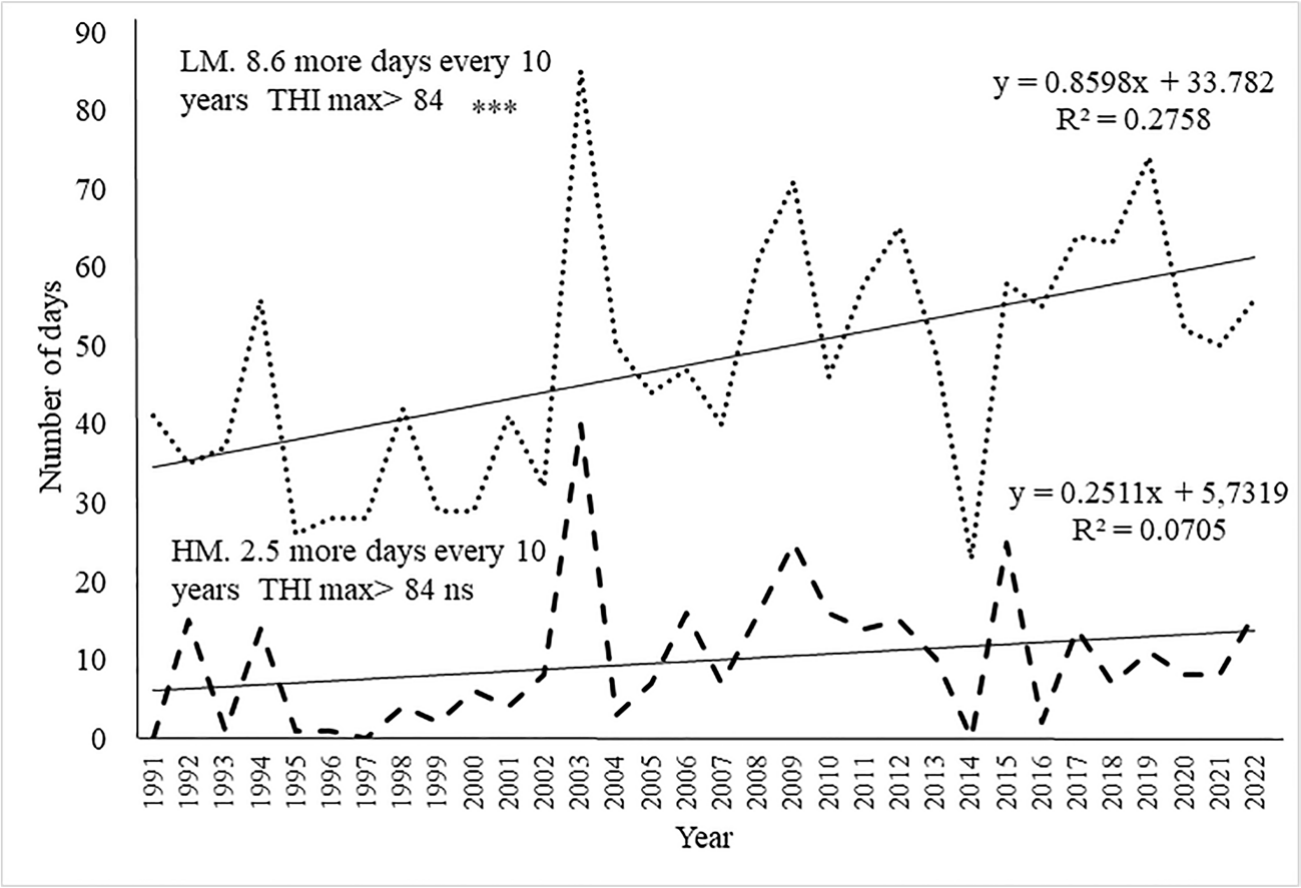
Number of summer days with outdoor spatialized THImax > 84 (risk class 4) in the period 1991–2022. LM Borgo S. Lorenzo, point line; HM Firenzuola, dashed line. *P <0.05, **P <0.01, and ***P < 0.001; n.s, not significant.

Considering instead the maximum daytime risk threshold (spatialized THImax > 84; class 4), the upward trend was statistically significant (*P* < 0.001) in LM with 8.6 more days every 10 years, while it was not in HM (Fig. 5).

### Welfare/heat stress conditions of dairy cows inside sheds and outdoor over the summers 2020–2022

In this section, only THI values derived from direct meteorological observation were used.

**Fig. 6:**
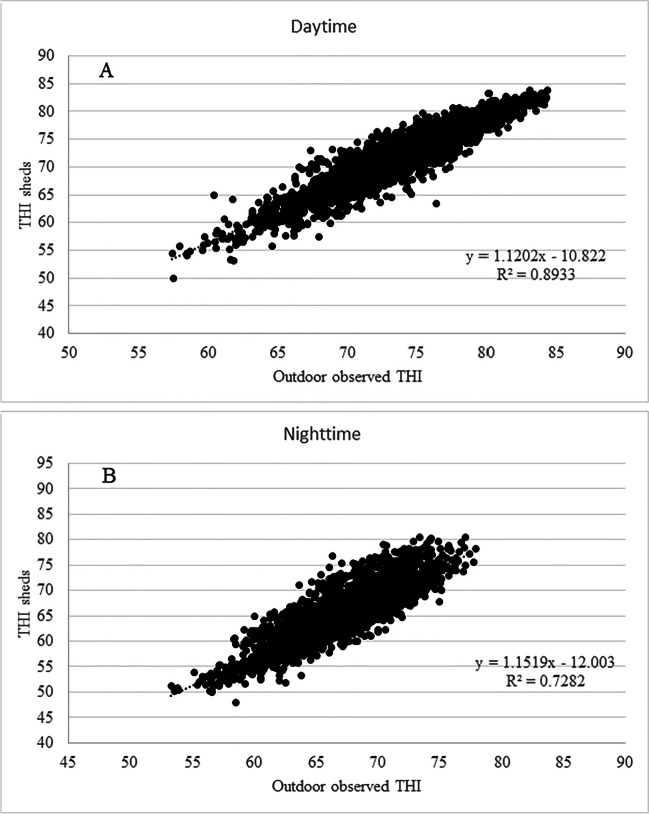
Correlation (*P* < 0.0001) between shed and outdoor observed hourly THI for LM daytime (**A**) and nighttime (**B**) in the summer periods 2020–2022

By comparing sheds and outdoor observed hourly THI by means of a linear regression analysis, a significant correlation between them (*R*^2^ = 0.92 in LM and *R*^2^ = 0.66 in HM) was observed (data not shown). Considering LM, the correlation was greater during daytime (*R*^2^ = 0.89, *P* < 0.0001) than in nighttime (*R*^2^ = 0.73, *P* < 0.001) (Fig. 6); similar results were also observed for HM even if with a lower correlation (data not shown).

**Fig. 7:**
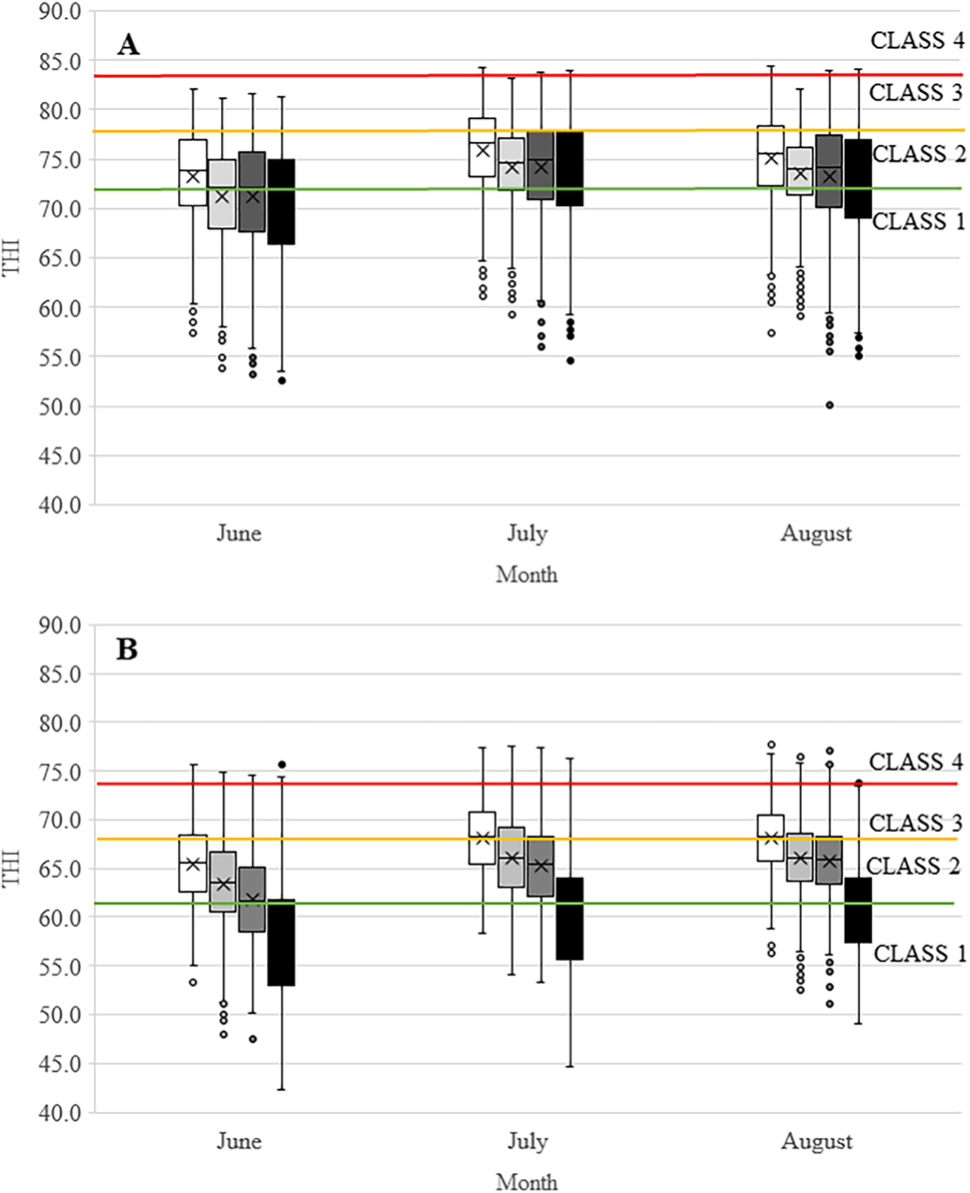
Monthly distribution of hourly THI during the daytime (**A**) and nighttime (**B**) in the summer periods 2020–2022. Box, values between the 25th and 75th percentile; X in the box, median; line in the box, mean; white box, sheds LM; light grey box, sheds HM; dark grey box, outdoor LM; black box, outdoor HM

In Fig. 7, the summer monthly frequency distribution of hourly THI outdoor and inside sheds, for HM and LM, is shown for daytime (Fig. 7A) and nighttime (Fig. 7B).

Considering daytime, the sheds in LM (box white) recorded, in all the summer months, the highest THI values compared to both the sheds in HM (box light gray) and the outdoor environments.

The highest shed THI values were recorded in July with mean and maximum values of 75.9 and 84.3 respectively, followed by the month of August (mean 75.0; max 84.4). As regards the HM sheds, the highest average values were recorded in July (74.2) with maximum peaks of 83.2. Considering the outdoor environments, the differences between areas were less, but with LM values slightly higher than HM ones, with the exception of July when values are very similar. Also, during nighttime, the LM sheds were uncomfortable than in HM, during all summer months, with the highest values in July and August (mean 68.6 and 68.3, respectively). Mean value for HM were 66.6 in August and 66.4 in July. Greater differences between the two areas were observed outdoor during the nighttime when the HM tend to cool down much better than LM.

Table 2 shows the monthly percentage frequencies of each risk class during the daytime period (07:00 am–20:00) in LM and HM both in the sheds and outdoor environments.

**Table 2:** Percentage of hours of the daytime period for each THI risk class

Daytime (07:00–20:00)	Farm areas	THI risk classes			
		1	2	3	4
		THI ≤ 72	72 < THI ≤ 78	78 < THI < 84	THI ≥ 84
June	LM shed	37.5	47.3	15.2	0.0
	HM shed	53.7	42.2	4.0	0.0
	LM outdoor	49.7	42.4	7.9	0.0
	HM outdoor	78.3	18.6	3.2	0.0
July	LM shed	17.7	46.1	35.9	0.3
	HM shed	26.8	57.1	16.1	0.0
	LM outdoor	30.5	45.7	23.8	0.0
	HM outdoor	61.8	22.1	16.1	0.0
August	LM shed	24.0	48.3	27.4	0.2
	HM shed	29.7	61.4	8.8	0.0
	LM outdoor	35.1	45.2	19.7	0.0
	HM outdoor	66.7	22.2	11.1	0.1

In outdoor environments, during the daytime period in June, the HM had most of the hours with no risk (class 1); instead, in the LM, only about half of the hours (49.7%) were risk-free. In July and August, the hours at risk significantly increased, in LM, were 69.5% and 64.9% respectively, while in HM, the risk-free hours continued to prevail (July 61.8 %, August 66.7%). The two highest risk classes (3 and 4) were highly more frequent in the LM; in particular, in July were 23.8%, compared to 16.1% in HM. Similar behavior was also found in August (19.7% in LM and 11.2% in HM).

Overall, in both areas, the situation was worse in the shades, with confirmed greater frequency of high classes (3 and 4) in LM. In particular, in August, the hours with class 3 or 4 was overall about three times higher in LM (27.6%) than in HM (8.8%). Furthermore, the class 4 has never occurred in the HM shades during the entire summer period.

Also, during the nighttime, differences emerged, both in the shades and outdoor environments (Table 3).

**Table 3:** Percentage of hours of the nighttime period for each THI risk class

Nighttime (21:00–06:00)	Farm areas		THI risk classes		
		1	2	3	4
		THI ≤ 62	62 < THI ≤ 68	68 < THI < 74	THI ≥ 74
June	LM shed	20.7	52.1	26.5	0.8
	HM shed	41.4	42.9	15.6	0.1
	LM outdoor	52.8	36.9	10.0	0.2
	HM outdoor	84.0	10.2	5.0	0.8
July	LM shed	7.0	39.9	48.4	4.7
	HM shed	16.6	49.9	32.2	1.4
	LM outdoor	24.1	47.8	27.0	1.1
	HM outdoor	77.0	15.7	5.5	1.8
August	LM shed	4.0	42.7	49.2	4.1
	HM shed	13.1	55.2	31.0	0.6
	LM outdoor	15.6	56.8	25.1	2.5
	HM outdoor	76.0	17.2	6.8	0.0

In the outdoor HM environment, during the summer period, about 78% of the hours were not at risk (class 1) against only about 30.8% in LM. In sheds, in LM, about 53% of the hours in July and August were in the two highest risk classes (3 and 4), with 4.7% in July and 4.1% in August in class 4. In HM sheds, on the other hand, the frequency of the two maximum risk classes was much lower during all summer months.

The animal data recorded by radio-collars put in evidence of different behaviors linked to the THI increase (Table 4).

**Table 4:** Variation of behavioral parameters with the increase of each THI point (daytime and nighttime)

	Increase in THI-D				Increase in THI-N			
		*P*-value	*F*	FxTHI		*P*-value	*F*	FxTHI
Eating	− 0.25	< 0.001	***	***	− 0.21	< 0.001	***	***
Rumination	− 0.16	< 0.001	***	***	− 0.14	< 0.001	***	***
Inactivity	+ 0.39	< 0.001	***	***	+ 0.34	< 0.001	***	***

*THI-D* diurnal temperature–humidity index. *THI-N* nocturnal temperature–humidity index, *F* farm effect; *FxTHI* interaction between farm and THI, *P*-*value*, significance value for the effect of increasing THI, *n.s* not statistically significant

*P < 0.05

**P < 0.01

***P < 0.001

**Fig. 8:**
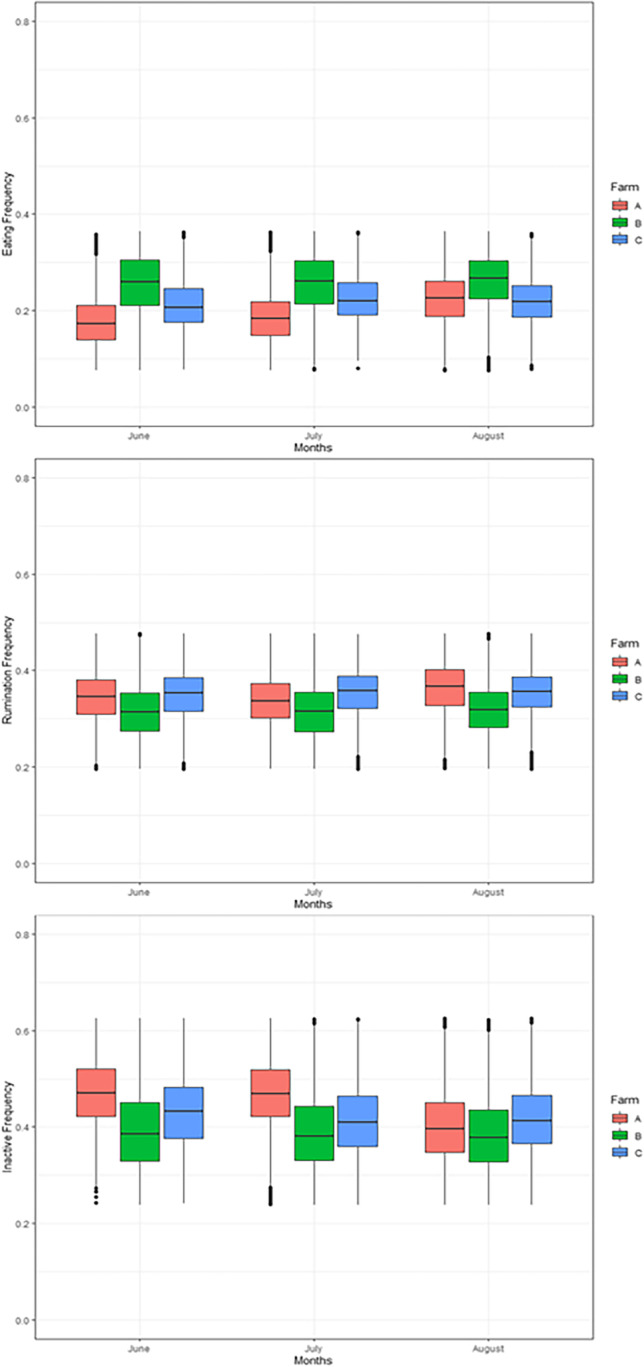
Descriptive analysis of the variation in behavior in the 3 farms over the 3 months of the study. Box, values between the 25th and 75th percentile; line in the box, mean

In particular, it is denoted how the animal’s behaviors are associated with increases in THI both during the day and at night. On average the animals showed a decrease in feeding (− 0.25 and − 0.21 THI-D and THI-N, respectively) and rumination (− 0.16 and − 0.14 THI-D and THI-N respectively) frequency and an increase in general inactivity (+0.39 and +0.34 THI-D and THI-N, respectively) for each THI point of increase. As regards the behavior of the animals in each individual farm (F), although the trend of the values with respect to the increase in THI was similar, the data recorded significant differences as shown in the table 4 and as can be extrapolated from the descriptive analysis of the Fig. 8.

Figure 8 shows a different behavior in the three farms, with the Farm B located in the HM showing fewer animal stress and less variable behavior over the months. Farm A showed lower feeding values especially in the periods of June and July connected to an increase in animal inactivity.

## Discussion

The use of outdoor spatialized data made it possible to obtain the maximum daily THI values of the last 30 years for two locations representative of the two study areas and for which long time series of data from weather station were not available. The data analysis highlighted a significant trend in the increase of heat stress conditions (increase in THImax values) in the summer period 1991–2022, in Mugello areas, more evident in LM despite HM. Our results confirm that of Polsky and von Keyserlingk (2017), according to which the number of days where THI exceeds the comfort threshold (> 72) is increasing in the northern United States, Canada, and Europe. This suggests a potential aggravation of the heat stress conditions for farmed animals.

However, as shown by Segnalini et al. (2011) for the period 1951–2007, THI increasing trend in Mediterranean Basin were not homogeneous in all geographic areas. In particular, in our study, the increase in THImax values (about 1 more per decade in both areas) was coherent with the average temperature increase per decade in Europe between 1991 and 2021 according to the Copernicus Report (2022). This confirms that the Mediterranean region is one of the most responsive areas to climate change and was identified as a major “hot-spot” based on global climate change analyzes (Todaro et al. 2022). In support of this, a rather recent study (Morabito et al. 2017) highlights how in the period 1980–2015, the frequency and intensity of heat waves significantly increased in the main cities of the Mediterranean Basin. The significant correlation between the outdoor spatialized THI and the outdoor observed THI data during the three summer seasons of the study confirms the adherence of the spatialized data with the measured one also on the study areas, thus allowing to strengthen the meaning of increase trend found in THI values in Mugello. Furthermore, the THI values inside the sheds showed a high correlation with the outdoor observed THI, substantially confirming what was identified by Chamberlain et al. (2022) in southern England cowsheds where, during the summer season 2021, a Pearson correlation coefficient between 0.94 and 0.98 was observed.

During all the summer months, the THI inside the sheds was higher than that measured outdoors in both areas, confirming what was identified by Shock et al. (2016) which highlighted an underestimation (1 unit less of THI) of the conditions of heat stress in 48 stables in Ontario (Canada) using outdoor data. In addition, the uncertainty of the measurement must also be take into account, as shown by Hempel et al. (2018).

Shock et al. (2016) also highlighted a great variability in the THI values among the 48 cowsheds monitored in Canada, variability which, according to the author, can be attributed to the different characteristics of the sheds, in particular their orientation and presence within adaptation systems such as fans. This consideration, although the number of sheds in our study was decidedly lower, also appears to be valid for Mugello where some sheds have sprayers and fans while others have none. The sheds construction materials also have their influence, concerning this a recent study conducted in the Czech Republic (Kic 2022) showed as older brick cowsheds, used after reconstruction and modernization in many cases, for housing certain groups of cows, such as dry cows, or before and after calving, have a better heat storage. This is reflected in a good attenuation of indoor air temperatures and a shift of the highest temperatures by several hours.

The study revealed that in Mugello, and in particular in LM sheds, more than 70% of daytime hours during the summer period were characterized by heat risk conditions (THI > 72) for livestock with potential consequences both for their health and productivity. Pinto et al. (2020) reported that environmental conditions already under a THI between 70 and 74 can produce a potential heat stress for cattle. A decline in the thermal gradient between an animal and its environment due to heat stress (high THI value) compromises the loss of metabolic heat and contributes to heat load (Kaufman et al. 2018). Numerous studies have shown that THI above 72 causes a decreased milk production by 21% and dry matter intake by 9.6% (Herbut et al. 2018; Bouraoui et al. 2002).

As regards the nighttime, about 90% of the hours in the LM and 77% in the HM were characterized by THI higher than the first risk threshold (THI > 62) with potential widespread conditions of thermal heat stress for animals. Leliveld et al. (2022) had realized a study in cow farms in North Italy during 2018–2019, and they showed how high THI during nighttime had a significant negative effect on the lying duration and cows rest more while standing to improve evaporative cooling. This demonstrates the importance of continuous monitoring of the microclimate inside the sheds during the summer, even during the nighttime.

Analyzing the influence of climatic parameters recorded, especially inside the stables, on animal behavior, although a fairly short period of study was taken and for this reason characterized by limited climatic variations, it can be seen that the increase in THI involves variations in the three behaviors analyzed, highlighting trends that lead to possible situations compromising welfare and production. In fact, the decrease in feeding and rumination combined with the increase in inactivity lead to imbalances in the biological and physiological mechanisms of the animal as reported by many works (Grinter et al. 2022; Tao et al. 2020; Soriani et al. 2013) resulting in health and milk production consequences. The evaluation of the influence of nocturnal THI was also important, which showed that the lack of a restorative period for the animal can lead to an increase in stress due to the heat. Many studies report that the effect of heat stress in recent years is due precisely to the increase in THI even during the night, leading to a lack of possibility for the animal to recover during the 24 hours of the day (Vizzotto et al. 2015; Silanikove et al. 2009). The use of precision zootechnical tools, together with an adequate construction of the stable based on its location, can help mitigate the effect of heat stress. Many studies report how the location and correct orientation of the structures can affect the environmental condition (Polsky and von Keyserlingk 2017; Boyu et al. 2020). The values reported in the study demonstrate how farms located in different positions are conditioned by different environmental factors, impacting on animal health. In fact, as the authors expected, different managements or farm locations, although the trends of behaviors variation may be similar, can generate different conditions and therefore different values of these. The use of technologies such as those used in the present work, capable of recording various data of the animal in real time, can help the farmer in the continuous individual monitoring of the animals to prevent the onset of stress problems. In addition to these, the technological implementation of the structures, through instruments for the mitigation of problems related to heat stress, has proved to be valid strategies to combat the problem (Becker and Stone 2020; Almuhanna et al. 2021).

The main strength of this study is that microclimate monitoring in Mugello has highlighted how heat stress conditions during summer seasons (high THI values) now also occur in areas (inner Apennine valleys) where until a few years ago this problem did not have to be faced. The most marked heat stress conditions were recorded in indoor environments (sheds) frequented by the animals, thus highlighting the need to adopt adaptation strategies in the sheds to counteract the climate change.

Furthermore, the significant correlation between the outdoor observed THI and the sheds THI could allow to estimate the heat stress conditions inside the sheds even using outdoor data (albeit with a certain margin of uncertainty due to the many variables involved). In consideration of this, the implementation of a forecasting chain to predict the outdoor THI values, by means of meteorological model, could represent an important tool for risk estimation even inside sheds. However, the uncertainty between the weather data obtained from nearby weather stations and the climate inside the barns has to be taken into account, as well as the errors that can occur when using regression models to predict THI in barns (Mylostyvyi et al. 2020).

Knowing in advance the potential heat stress conditions could allow the farmer to deal with them more effectively, for example, by varying the animals’ feed in advance or in any case activating other possible mitigation strategies.

The main limitation of this study was represented by the small sample of cow sheds used which, moreover, are probably not well representative of the construction characteristics of the sheds (building materials and presence of fans or sprayers) located in the study areas. Despite this, the correlation between the THI in the sheds and the outdoor is however high and confirmed the results of other studies. In addition, data relating to animal behavior (monitoring with collars) and milk production collected during the study have not yet been processed and as soon as they are available they will be integrated to assess the impact of heat stress conditions on the animal performance. We also intend, in the coming months, to increase the study area to identify new dairy cow farms available to carry out new monitoring during the next summer seasons.

## Conclusions

The results showed that there was a direct interaction between the THI trends and the animal’s behaviors which are most used to monitor health status and anticipate production losses. In particular, how increases in THI have led to a reduction in the frequency of behaviors linked to feeding and an increase in inactivity. This also highlighted how the application of technological solutions can help the farmer in managing his herd. The characterization of the local climatic conditions thanks also to the correlation between external and internal data of the sheds can help to monitor more precisely the possible onset of stress conditions. Another useful adaptation tool for breeders could be the use of forecasting models able to provide information on the expected THI a few days in advance as well as an estimate of the potential associated loss of productivity.
